# Exploratory research on cognitive fluency design for the older adults through Nostalgia-based empowerment

**DOI:** 10.1007/s40520-026-03396-2

**Published:** 2026-04-11

**Authors:** Yingjia Zhou, Xianhua Sun

**Affiliations:** College of Art and Design, Nanjing Forestry University, Nanjing, 210037 China

**Keywords:** Cognitive fluency, Aging-friendly design, Nostalgia empowerment

## Abstract

**Background:**

As global aging intensifies, issues in age-friendly cultural consumption design have become increasingly apparent. Existing age-friendly designs primarily focus on simplifying functions while neglecting cognitive fluency, resulting in the current dilemma of “high demand-low adoption.”

**Purpose:**

To systematically validate the impact of cognitive fluency design, empowered by nostalgia, on age-friendly cultural consumption through a three-dimensional cognitive-physiological-psychological framework.

**Methods:**

Extensive literature searches and data collection were conducted across electronic databases including Web of Science, PubMed, and CNKI. This was followed by field research and interviews using questionnaire surveys. Findings were further refined through data analysis to advance research on cognitive fluency design for aging-friendly.This study is exploratory in nature, with a relatively small sample size, and the conclusions primarily provide preliminary reference and ideas for subsequent related research.

**Results:**

Findings demonstrate that in terms of emotional activation, nostalgic design effectively triggers pleasant feelings and enhances cognitive fluency among older adults. This approach yields multifaceted benefits for their physical and mental well-being, with particularly significant effects on orientation, memory, and fluency. This intervention pathway is crucial in research on cognitive fluency in aging.

**Conclusion:**

Cognitive fluency design is the key variable in overcoming the ‘last mile’ of age-friendly adaptation, and it needs to be supported by nostalgia-empowerment strategies to aid contemporary age-friendly design.

## Introduction

We have entered an era of population aging [1]. Existing research on aging-friendly design primarily focuses on engineering technology and functional optimization, with an emphasis on physical environments or product design. It severely lacks a user perspective, particularly that of the older adults group [2]. Within the field of cognitive fluency research, only scattered studies have addressed the cognitive abilities of the older adults group and related interventions, and a systematic theoretical framework has not yet been established.

Cross-cultural studies have shown that the older adults generally experience nostalgic emotions, which exhibit a certain degree of stability. Older individuals are more susceptible to cultural influences, so they focus more on the past than younger people, and such emotions affect their lives [3]. Meanwhile, nostalgia therapy has been proven to bring positive emotions to the older adults group [4], enhancing their self-identity and adaptability to current life through nostalgia and memories. Against this background, in-depth exploration of the mechanism by which nostalgia therapy affects the cognitive fluency of the older adults group and the construction of a scientific aging-friendly design system hold significant theoretical value and practical significance.

Given the aforementioned research context and practical challenges, this study aims to construct a cognitive-physiological-psychological three-dimensional framework. This study sought to validate the impact of nostalgia-enhanced cognitive fluency designs on older adults, determining whether such designs can effectively trigger positive emotions, such as pleasure, in this demographic, thereby enhancing their cognitive fluency levels. Revealing the intrinsic relationship between nostalgia therapy and cognitive fluency, this study aims to fill the gap in existing research regarding the cognitive domain of older adults individuals.

The scope of this study is primarily defined in the following three aspects: First, the research subjects are individuals aged 65 and above, covering different genders, educational backgrounds, and cultural contexts. Groups with severe cognitive impairments or mental illnesses are excluded to ensure the representativeness of the research sample and the validity of the data. Second, the research content focuses on the correlation mechanism between nostalgia and cognition, with an emphasis on exploring the impact of nostalgia design elements on the cognitive fluency of the older adults group. Studies on the impact of other intervention methods on cognitive fluency are not included for the time being. Third, the research scenario is limited to field studies in local communities within the country.

This study employs a multi-stage, multi-method mixed-methods strategy. Phase Two involved empirical research combining questionnaires and field interviews. The questionnaire employed anonymous sampling targeting older adults residents in Changzhou, China, to gather data on their nostalgic preferences and needs regarding songs. Simultaneously, semi-structured interviews were conducted with 20–30 older adults participants exhibiting diverse characteristics. These interviews guided participants through nostalgic emotional experiences and questionnaire responses, enabling an assessment of overall cognitive fluency to supplement survey data. The third phase involved data analysis and research interpretation. SPSS 27.0 and other software will be used to perform descriptive analysis and t-tests on the survey data. Integrating these findings with the literature review will refine the study of cognitive fluency in older populations within nostalgic contexts, ensuring scientific rigor and feasibility.

In conclusion, this study focuses on the nostalgia empowerment strategy and systematically explores the association mechanism between nostalgia therapy and cognitive fluency in the older adults group through multiple research methods. The research findings are expected to enrich interdisciplinary studies of aging-friendly design and cognitive psychology, provide new perspectives and ideas for academic research in related fields, and offer beneficial solutions for aging-friendly design practices, thereby helping to improve the quality of life and well-being of the older adults group.

## Theoretical framework

### Cognitive fluency

Cognitive fluency refers to a composite construct encompassing both the efficiency of information processing and subjective ease during cognition. Chuangchai’s study of aging-friendly design in Thai public facilities revealed that contemporary aging-friendly design predominantly addresses physical accessibility barriers while largely overlooking cognitive barriers [5].The findings indicate that in intelligent systems, users’ sense of familiarity—rather than novelty—effectively drives their willingness to use the systems, demonstrating that “nostalgic” elements serve as “familiar anchors” and reduce cognitive load [6].

### Nostalgia therapy

The term “nostalgia therapy” first emerged in geriatric psychiatry. It was originally proposed by Butler (1963), who, based on Erikson’s psychosocial development theory, suggested that life review can occur at any stage of life. He also noted that nostalgia therapy has a special role for the older adults group, helping older adults achieve self-integration and identity, and emphasized the importance of nostalgia in assisting older adults to adapt to their own aging process. On one hand, nostalgia therapy enhances self-identity through recalling the past and helps individuals better adapt to current life, thereby promoting physical and mental health. On the other hand, during its necessary interpersonal interaction process, it not only fosters interpersonal communication but also drives social acceptance. With low implementation costs, minimal side effects, and high convenience, nostalgia therapy has become an important means of older adults care abroad. Additionally, it has positive implications for treating dementia patients, helping to reduce the situation of polypharmacy.Previous studies have successfully implemented non-pharmacological nostalgia therapy in older adults communities through multiple product field practices [7].

### Research gaps and innovations

This study addresses three key limitations.

Theoretically, the study focuses on the cognitive fluency model, transcending the traditional engineering perspective of external factors. Methodologically, it combines cross-cultural contextual experiments to highlight the biological efficacy of nostalgic emotional experiences. Simultaneously, it integrates theory with practice, complementing offline surveys with online data.

### The older adults population

Aging is a natural, complex, and heterogeneous process that affects every aspect of human life [8]. It is not only associated with physiological decline but also brings challenges in the emotional and cognitive domains [9].

First, physiological decline poses significant cognitive challenges to the older adults. Factors such as diminished perception, weakened attention, and slowed information processing directly reduce cognitive efficiency during audio-visual experiences [10]. These cognitive experiences, in turn, affect emotions, leading to feelings of diminished autonomy and loss of control over daily life [11, 12].

Second, cognitive limitations emerge as memory capacity declines steadily with age in older adults. Memory impairment contributes to usage errors in up to 58% of cases. Psychologically, this triggers feelings of displacement, such as emotional alienation and emptiness when familiar devices or environmental elements are replaced, alongside technophobia, where fear of operational errors leads to avoidance of all modern technology.

These factors significantly impact the quality of life of the older adults population [13]. Scholars and experts strongly advocate research into appropriate intervention measures to support the healthy development of older adults [14].

### An exploration of cognitive abilities grounded in nostalgic sentiments

First, cultural identity is primarily expressed through emotional responses. Studies have revealed that nostalgia effectively improves mental health among older adults. By employing situational healing, it avoids the negative side effects of medication while providing spiritual pleasure to the audience.

Regarding emotional regulation, nostalgia assists individuals in managing their emotions, alleviating anxiety and stress, and enhancing emotional stability. By recalling past pleasant experiences, people can derive emotional comfort and fulfillment, thereby improving their mental well-being. When individuals reminisce about joyful memories, the brain’s reward system activates and releases neurotransmitters, such as dopamine, to induce feelings of pleasure and satisfaction.

In cognitive processing, nostalgia involves self-reflection and autobiographical memory, promoting an understanding of identity and life experiences. This process of introspection and recollection aids in establishing self-identity, while boosting self-esteem and confidence. Long-term memory is a key characteristic of cultural consumption among the older adults. Nostalgia-based designs circumvent the learning barriers associated with novelty, significantly enhancing the willingness to consume culture. By fostering a sense of belonging, they drive a sustained engagement.

Enhancing cultural background compatibility can effectively reduce cognitive load among the older adults. Their sense of cognitive identity manifests in daily decision-making, closely tied to factors like cultural background and social support. Different cultural backgrounds shape distinct habits. Cultural backgrounds also exert significant influence on intergenerational communication. For instance, Chinese elders, often characterized by collectivism, and American elders, who tend to pursue individualism, exhibit distinct differences in the frequency of social interactions with their children. This cultural influence can even be transmitted to the next generation [15]. Therefore, gaining a deeper understanding of the cultural background needs of different groups can enhance the cognitive fluency of cultural consumption among the older adults.

Furthermore, some modern age-friendly products raise significant privacy and ethical concerns. Data indicate that most smart toilet seats with urine detection capabilities are connected to children’s smartphones, yet seniors often express a low willingness to share such data. This necessitates the identification of an optimal solution that balances technological convenience and privacy protection.

## Methodology

### Research ethics

This study was conducted in conventional educational or workplace settings and falls under the category of low-risk social survey research. The study adheres to relevant academic standards and applicable ethical oversight mechanisms. The questionnaire clearly states the research objectives, data usage, and privacy protection measures at the outset. Participants provide informed consent by voluntarily submitting the questionnaire, which constitutes agreement to participate in this study and authorization for their data to be used in research analysis. All collected data undergoes rigorous anonymization. Throughout the entire process of data storage, usage, and analysis, participant privacy and information security are safeguarded to effectively protect participant rights and interests.

### Research design

#### Identifying nostalgic elements

Nostalgic elements encompass a wide spectrum. Selection could be based on literature review and interviews. For experimental convenience, music and visual media were chosen as primary nostalgic elements. These elements typically evoke strong personal memories, with preferences varying by age group. Therefore, multiple factors were considered when defining the scope of elements:

1. Selecting popular music from 1964 to 1994 aligns with the youth-to-middle-age memories of current 65-80-year-olds. This period is crucial for emotional resonance between musical memories and nostalgic feelings. Additionally, five popular songs unfamiliar to the older adults cohort were chosen to serve as a contrast to nostalgic music during the study.

Nostalgic Playlist

1978 Dede Ma - “Beautiful Grasslands, My Home”.

1979 Li Guyi “Longing for My Hometown”.

1980 Jiang Dawei “Where Peach Blossoms Bloom”.

1981 Zhu Fengbo “Golden Shuttle and Silver Shuttle”.

1982 Zhu Mingying “Ocean, My Hometown”.

1983 Ji Xiaoqin “Song of the Yangtze River”.

1984 Li Guyi “Unforgettable Tonight”.

1985 He Jiguang “Tattered Shoes, Tattered Hat”.

1986 Fei Xiang “A Blaze in Winter”.

1987 Chen Li “Wrongly Furrowed Brows”.

1988 Jiang Dawei “Dare I Ask Where the Road Lies”.

1989 Cui Jian “The Fake Monk”.

1990 Mao Amin “Longing”.

1991 Na Ying “Mountains Stand Still, Waters Flow On”.

1992 Mao Ning “The Waves Still Roll”.

1993 Yang Yuying & Mao Ning “Heart Rain”.

1993 Li Chunbo “Little Fang”.

1994 Yang Hongji “The Rolling Yangtze Flows Eastward”.

Non-Nostalgic Playlist

2021 Huang Xiaoyun “Starlit Seas”.

2021 Faye Wong “As Desired”.

2022 Lei Jia “Human World”.

2023 Huang Qishan & Xilinai Gao “Mother and Daughter”.

2024 Zhou Shen “Little Happiness”.

2. Fifteen senior students were randomly selected for interviews. They listened to two songs from the nostalgic playlist and one song from the non-nostalgic playlist, played randomly. The participants completed a nostalgia questionnaire, recording their familiarity, nostalgic feelings, and enjoyment levels for each song.

**Fig. 1:**
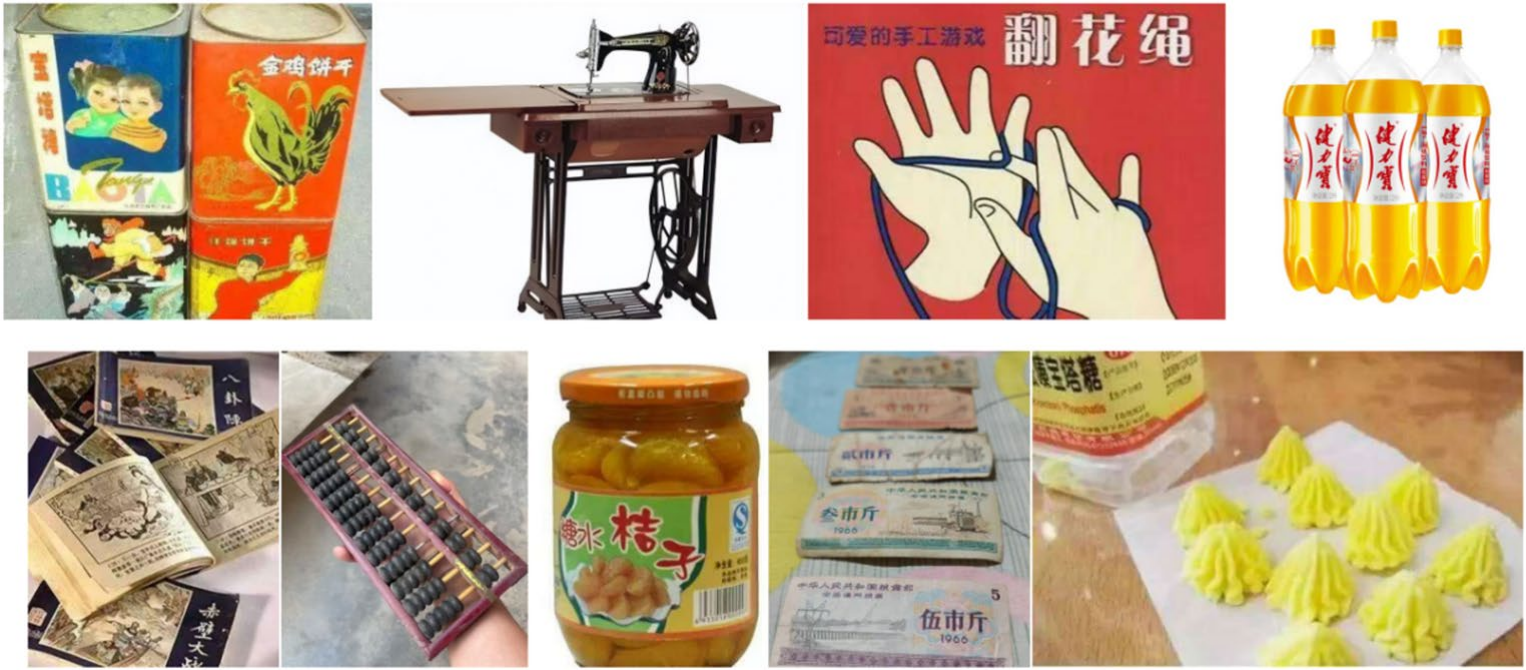
.xxx

For nostalgic visual elements, the selection primarily focused on displaying images and videos of vintage objects and life scenes. These included sewing machines, cassette tape recorders, black-and-white televisions, tin thermos flasks, honeycomb coal stoves, and old movies, all sourced from the Internet (Figure1). In addition, some modern appliances and contemporary life scenes were collected to provide contrast.

### Community field research

**Fig. 2:**
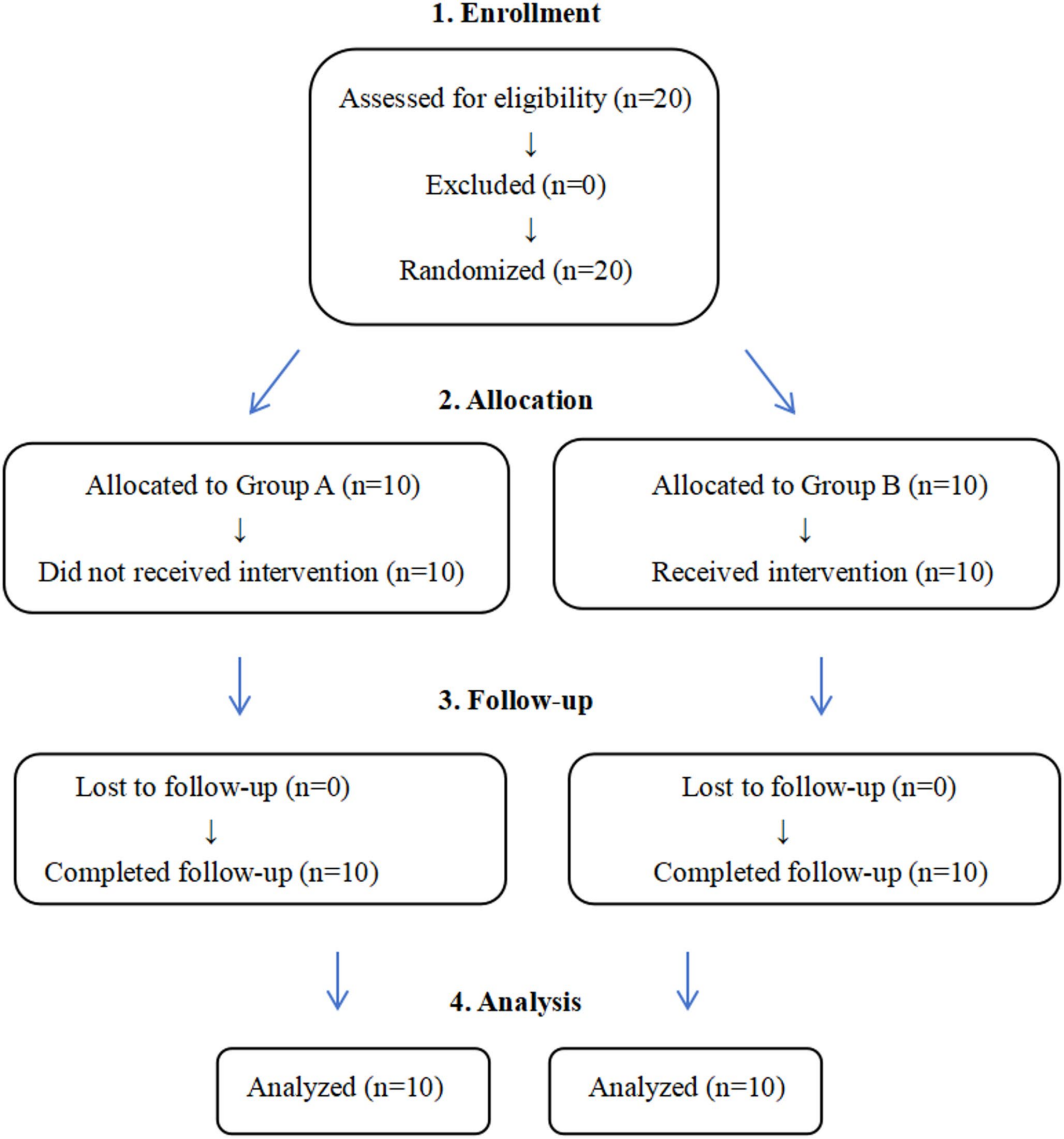
.xxx

Offline interviews and multiple questionnaires were administered simultaneously. Two testing sessions were held in the community, each lasting 25–30 min. The participants were divided into two groups for comparison, with each group comprising 10–15 people.Grouping was conducted randomly within the same location and time setting. Evaluators did not blind the intervention status, as the intervention characteristics were highly conspicuous and difficult to conceal. (Fig. 2)

**Fig. 3:**
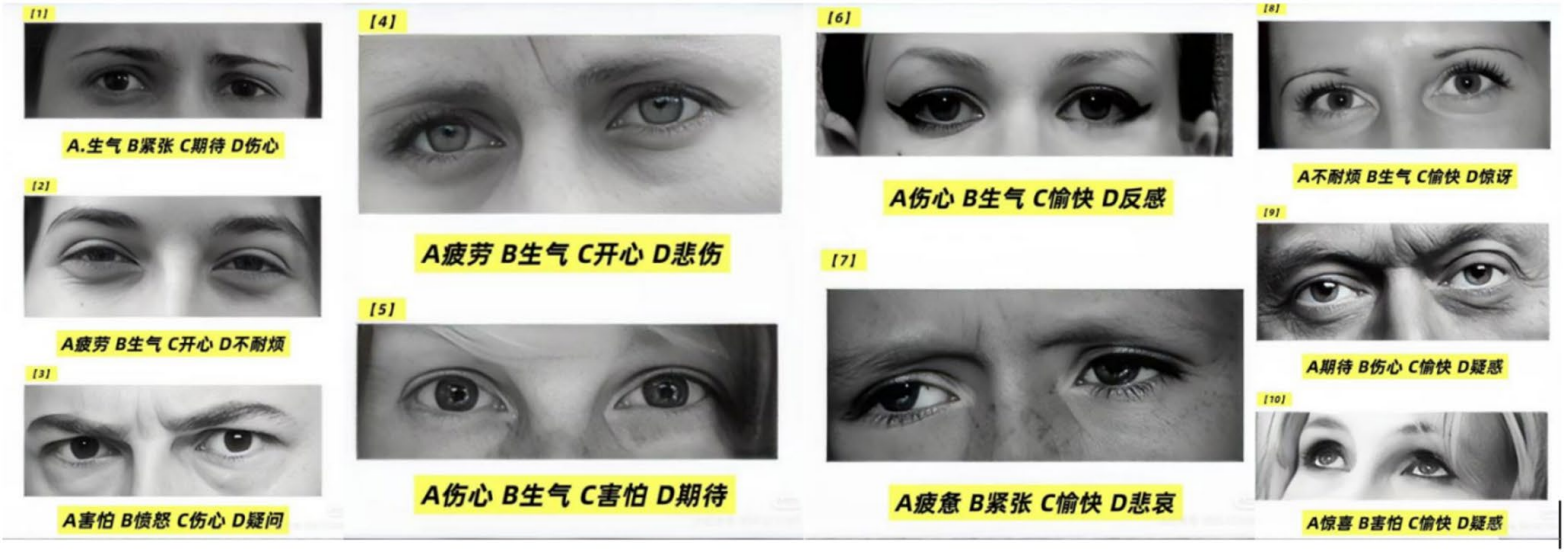
.xxx

**Fig. 4:**
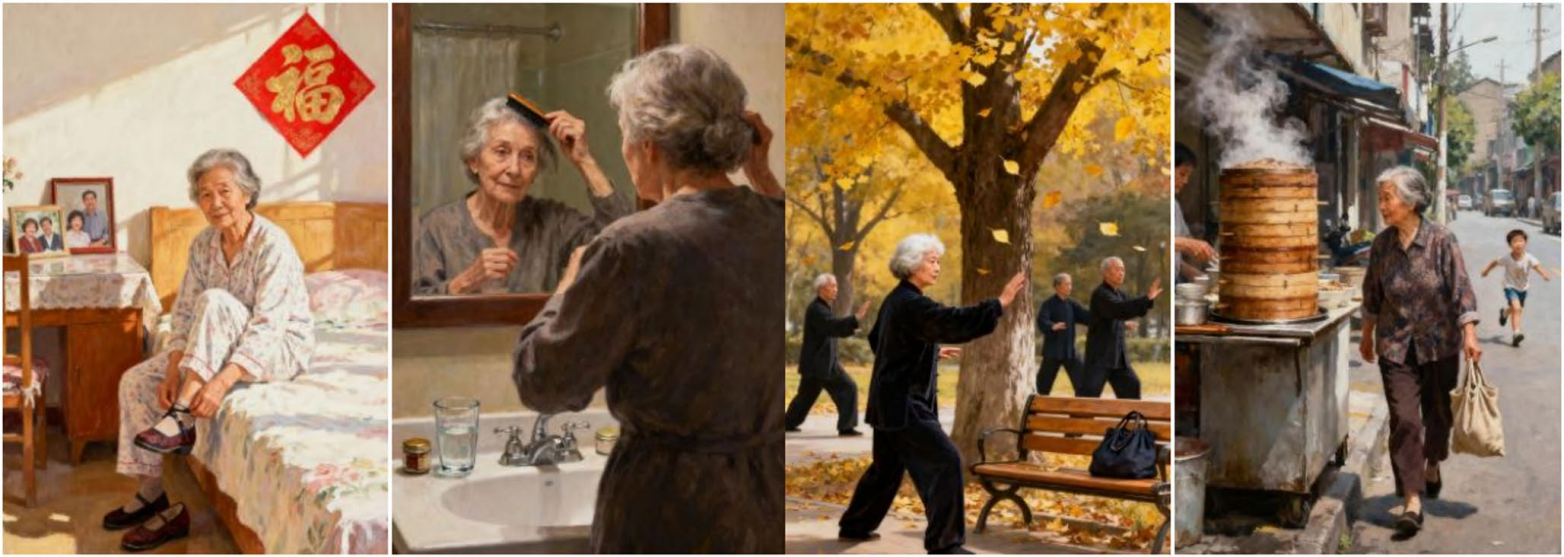
.xxx

Group A (10 participants):

A structured protocol was followed with the following testing sequence:

1. 5-minute icebreaker session: Participants were welcomed and a whiteboard was used to discuss the date, current weather, location, and current events to quickly build rapport.
2. 15–20 min of core activities: Experience and perceive non-nostalgic music, followed by sequential completion of the eye-tracking test (Fig. 3), picture sequencing task (Fig. 4), simple story retelling with related questions. Concurrently, assess overall cognitive function based on task performance and on-site status, including attention, orientation, memory, language ability, fluency, visuospatial skills, and problem-solving across multiple domains. Adjustments may be made at any time according to the participant’s specific condition.The eye-reading test employed in this study is a modified version adapted from the “Reading the Mind in the Eyes” test (RMET).
3. 5-minute wrap-up: Thank each participant for their contribution, remind them of the date of the next session, and conclude the session [16].

Group B (10 participants):

Structure identical to Group A, with the addition of nostalgia-inducing elements during the test. Testing process:

1. 5-minute introduction: Welcome participants and use a whiteboard to discuss the date, current weather, location, and recent events to quickly build rapport.
2. 15–20 min core activity. Key steps include incorporating nostalgia-inducing interventions, such as participants’ experiences with nostalgic songs, visuals, or environmental elements, and guiding them to recall and discuss positive personal memories. Subsequently, the participants completed the following tasks in sequence: eye-reading test, picture sequencing task, and simple story retelling with related questions. Concurrently, an overall cognitive function assessment was conducted based on task performance and on-site observations. This evaluation covers multiple domains, including attention, orientation, memory, language ability, fluency, visuospatial skills, and problem-solving. The assessment can be adjusted in real time according to each participant’s specific condition.
3. A 5-minute wrap-up session concluded the training. This included thanking each participant for their contribution, reminding them of the schedule of the next activity, and bidding farewell.

Early morning, Grandma sat on the edge of her bed in her pajamas, bending down to put on her shoes. A “Fu” character sticker adorned the headboard, and family photos rested on the nightstand.

She made her way to the vanity, combing her hair in the mirror. A water cup and small jar sat on the countertop.

Grandma arrived under a tree in the park, practicing Tai Chi with several older adults friends. Yellow leaves drifted down from the branches, and her cloth bag rested on a nearby bench.

Carrying her bag, Grandma walked past street food stalls where steam rose from steamer baskets. Not far away, children ran along the roadside.

Three accompanying cognitive questions (corresponding to story details and logic).

Which action did Grandma perform before leaving home? (Assessing sequential memory: putting on shoes → combing hair)

What color were the leaves on the trees surrounding Grandma in the park? (Assessing detail memory: golden yellow/yellow)

What scene could Grandma observe while carrying her cloth bag past the breakfast stall? (Assessing scene association memory: children running/steam rising from the stalls)

### Sampling

Sample Source: The study was conducted in a local community in Changzhou, Jiangsu Province, using survey questionnaires and in-person interviews. A total of 36 questionnaires were distributed, with 35 valid responses collected (15 for Questionnaire a and 20 for Questionnaire b). The sex ratio was 2:3 (male to female). The age distribution comprised 28 participants aged 65–75 years and 7 participants aged 75–85 years.

Inclusion Criteria: Participants were aged ≥ 65 years, possessed adequate comprehension and communication abilities, and exhibited no severe behavioral symptoms likely to interfere with participation.

The exclusion Criteria: History of neurodevelopmental disorders, pre-existing intellectual disabilities, psychiatric disorders, or physical disabilities.

### Assessment measures

#### Two questionnaires were completed during the testing process

The first was the Nostalgia Scale [17], which was self-administered by participants while music was playing. It comprised six sections: name, age, sex, music familiarity, nostalgia level, and pleasure level. A 5-point Likert scale was used, with participants selecting a number from 1 to 5 for each response. Scores ranged from low to high: 1 indicated very unfamiliar, 2 indicated somewhat unfamiliar, 3 indicated neither familiar nor unfamiliar, 4 indicated somewhat familiar, and 5 indicated very familiar. Higher scores indicate greater familiarity with the music, stronger nostalgic feelings, and higher pleasantness. This questionnaire enables a preliminary assessment of the relationship between nostalgic elements and pleasure. As shown in Table 1.

During the actual interview process, considering that the older adults may be unfamiliar with specialized terms like nostalgia level or pleasure level, these concepts can be expressed in plain language. For example: “Have you heard this music before? Does it sound somewhat familiar, or is it new to you? Does this music bring back memories of the past? How does this music make you feel? Does it make you feel happy or joyful?” Respondents can rate their feelings on a scale of 1 to 5, where 1 indicates ‘I’ve never heard this before’ and higher scores indicate greater familiarity.” Additionally, interview strategies can be adjusted based on the specific circumstances of each participant. Scoring can be based on their written responses to ensure the validity of the ratings.

**Table 1:** a nostalgia scale

Table Name	Specific indicators	Measurement method	Reliability(Cronbach’s α)	Scoring (High score is better/Worse)	Operation
a nostalgia scale	Name	Self-filled			Play non-nostalgic music and images, videos
	Age				
	Gender				
	Familiarity		5-point Likert scale		
	Nostalgia				
	Pleasure				
a nostalgia scale	Name	Self-filled			Play nostalgic music along with pictures and videos
	Age				
	Gender				
	Familiarity		5-point Likert scale		
	Nostalgia				
	Pleasure				
Select one number from 1 to 5 for each response, with scores ranging from low to high as 1, 2, 3, 4, 5. (Where 1 indicates not at all familiar, 2 indicates somewhat unfamiliar, 3 indicates neither familiar nor unfamiliar, 4 indicates fairly familiar, and 5 indicates very familiar.)					

The second questionnaire was the B-Overall Cognitive Function Assessment Questionnaire [18, 19], this assessment scale, developed based on the MMSE and supplemented by literature review and synthesis, was refined to suit the needs of this study. It is completed by an observer while participants perform the eye-reading test and picture-sequencing task, covering seven domains: attention, orientation, memory, language, fluency, visuospatial ability, and problem-solving. A 5-point Likert scale is employed, where higher scores indicate superior overall cognitive functioning. As shown in Table 2

**Table 2:** B Global cognitive function assessment questionnaire

Age:		Gender:		Group A/B
Table Name	Specific indicators	Reliability (Cronbach’s α)	Scoring (High score is better/Worse)	Operation
B Overall Cognitive Function Assessment	Attention			
	Orientation			
	Memory			
	Language skills			
	Fluency			
	Visual-spatial skills			
	Problem-solving skills			
Select one number from 1 to 5 for each response, with scores ranging from low to high as 1, 2, 3, 4, 5. (Where 1 indicates not at all familiar, 2 indicates somewhat unfamiliar, 3 indicates neither familiar nor unfamiliar, 4 indicates fairly familiar, and 5 indicates very familiar.)				

#### The assessment includes two interactive activities to support the completion of the B-type global cognitive function questionnaire

Eye Expression Interpretation Test: Participants viewed 10 photographs of different actors’ eye expressions and identified the emotion conveyed through nonverbal cues. They selected the most appropriate option from four choices for each image. The final score was based on the number of correct answers, with higher scores indicating stronger emotional recognition ability.

Picture Sequencing Task: Participants were presented with story images in a random order. They must rearrange them, briefly narrate the entire story, and provide quick responses to related questions. The final score was the sum of the correct sequences and answers. A higher score indicates better cognitive states, such as memory and problem-solving abilities, as shown in Table 3.

**Table 3:** Questionnaire response log

Answer Record													
	1	2		3	4	5		6	7	8		9	10
Emotional Judgment Correct √													
Project			Answer Reference				Answer				√/×		
Story Sequencing			1243										
Question 1			Combing Hair										
Question 2			Yellow										
Question 3			Steamer Basket Child										
											Total Score		

### Data analysis

Comprehensive analysis of test results and data was conducted using SPSS tools, with visual representations created through charts and graphs to more intuitively demonstrate the relationships and changes among various factors.

## Results

### Descriptive statistical analysis

In this study, 20 valid questionnaires for the B-type Overall Cognitive Function Assessment were collected. Among them, 15 participants were female (75%), and 5 were male (25%), indicating a higher proportion of female respondents. The majority of respondents were aged 70–79 years, accounting for 70% of the sample. The specific details are illustrated in the Table 4 below.

The samples across different groups did not exhibit significant differences in terms of gender and other factors (p > 0.05), indicating that the different groups demonstrated consistency and no variation, making them suitable for experimentation. As shown in Table 5

**Table 4:** Participant information table

Item	Option	Frequency	Percentage
Gender	Male	5	25%
	Female	15	75%
Age	65–69 years old	3	15%
	70–74 years old	8	40%
	75–79 years old	6	30%
	80–84 years old	3	15%

**Table 5:** Participant grouping table

	CG	EG	χ²/t	
Gender			χ²=0.267	0.606
Male	3 (30.0)	2 (20.0)		
Female	7 (70.0)	8 (80.0)		
Age	73.8 ± 5.095	73.8 ± 3.967	t = 0.000	1

### Reliability analysis

As shown in the table, the reliability coefficients of the scale items are relatively high, indicating that the survey data are considered reliable (Table 6).

**Table 6:** Reliability statistics

Reliability Statistics		
	Cronbach’s Alpha	Cronbach’s Alpha Based on Standardized Items
Total	0.820	0.823

### Validity analysis

The KMO value exceeds 0.6, and the Bartlett’s sphericity test statistic shows a significance level of 0.000 < 0.01, indicating that the data possess good validity (Table 7).

**Table 7:** KMO and bartlett’s test

KMO and Bartlett’s Test		
Kaiser-Meyer-Olkin Measure of Sampling Adequacy.		0.663
Bartlett’s Test of Sphericity	Approx. Chi-Square	57.095
	df	21
	Sig.	0.000

### Differential analysis

Test scores (t = -2.25, *P* < 0.05) refer to the scores from the emotion judgment and story sequencing sections, comprising 14 questions totaling 14 points. The chart indicates significant differences between the two groups, with the intervention group showing higher mean scores overall, suggesting that the older adults group under the nostalgia intervention demonstrated superior performance.

Attention (t=-1.964, *P* > 0.05), language ability (t < 0.001, *P* > 0.05), and visuospatial ability (t=-0.372, *P* > 0.05), Problem-solving ability (t=-1.555, *P* > 0.05). The table shows no significant differences between the two groups in attention, language ability, visuospatial ability, or problem-solving ability under either nostalgic or non-nostalgic interventions.

Orientation (t = -3.539, *P* < 0.05), memory (t = -2.869, *P* < 0.05), Fluency (t = -3.536, *P* < 0.05). The table indicates significant differences in orientation, memory, and fluency between the nostalgia intervention and non-nostalgia intervention groups. older adults individuals in the nostalgia intervention group scored higher and performed better in these cognitive domains. Significant differences existed between the two groups, with the intervention group exhibiting higher mean scores.

The total cognitive function assessment score (t = -2.77, *P* < 0.05) represents the cumulative mean score across seven domains: attention, orientation, memory, language ability, fluency, visuospatial ability, and problem-solving ability. The intervention group showed significantly higher scores than the non-intervention group, indicating that the overall cognitive function of the older adults group was better manifested under the nostalgic intervention scenario.

An effect size ≥ 0.8 is considered large. Under the nostalgic intervention measures, the older adults group demonstrated large and statistically significant effect sizes in orientation, memory, fluency, and overall cognitive function, indicating that the intervention has practical significance (Table 8).

**Table 8:** Difference analysis

	group	M	SD	T	*P*	Cohen’sd
Test Scores	CG	6.800	1.135	-2.250	0.037	1.006
	EG	8.000	1.247			
Attention	CG	4.700	0.483	-1.964	0.081	0.878
	EG	5.000	0.000			
Orientation	CG	3.700	0.483	-3.539	0.002	1.583
	EG	4.500	0.527			
Memory	CG	3.300	0.675	-2.869	0.010	1.283
	EG	4.100	0.568			
Language	CG	4.500	0.707	<0.001	1.000	0.001
	EG	4.500	0.850			
Fluency	CG	3.800	0.789	-3.536	0.002	1.581
	EG	4.800	0.422			
Visual-Spatial Skills	CG	3.800	0.632	-0.372	0.714	0.166
	EG	3.900	0.568			
Problem Solving	CG	3.900	0.876	-1.555	0.137	0.696
	EG	4.400	0.516			
Total Score	CG	27.700	3.592	-2.770	0.013	1.239
	EG	31.200	1.751			

Differential analysis demonstrates that cognitive fluency in elderly groups improves and changes under nostalgic conditions. The baseline variables tested—gender and age—show no significant differences, with each participant’s test scores providing a direct reflection of their cognitive level. Furthermore, comparative analysis reveals that differences in attention, language ability, visuospatial ability, and problem-solving ability are not significant. These variations may be more closely linked to subjective factors such as individual conditions and cultural background. Conversely, orientation, memory, and fluency are influenced by nostalgic emotion intervention. Under nostalgic conditions, the mean values for these factors are higher, contributing meaningful insights to research on the cognitive levels of elderly populations in nostalgic contexts.

## Discussion

This study aimed to enhance the quality of life of the older adults by understanding the impact of nostalgia therapy on their cognitive levels. Building on an extensive literature review, experimental data were collected through face-to-face interviews and questionnaires. SPSS software was used to validate data reliability and conduct further analysis, thereby refining the research.

Numerous studies have indicated that cognitive processes are influenced by emotions, including attention [20], memory [21, 22], reasoning ability [23], and problem-solving skills [24], all of which are indispensable in daily life. Research findings have revealed positive emotions among older adults in nostalgic contexts. Nostalgia enhances feelings of comfort and security [25] and maintains healthy psychological states within specific timeframes [26]. The unique sense of reassurance that nostalgia provides to older adults is irreplaceable [27].

Nostalgic design triggers significant neural activity, stimulating older adults and regulating their physiological functions [28]. Existing research confirms that older adults recalling nostalgic memories exhibit autonomic nervous system inhibition accompanied by increased parasympathetic activity [29]. Nostalgic scents can alleviate depression and anxiety in the older adults [30], whereas nostalgic imagery enhances feelings of ease [31]. This fosters positive emotions, such as emotional resonance and pleasure, increases participation willingness, improves cognitive fluency, and suppresses systemic inflammation [32, 33].

Data analysis from this study indicates that cognitive abilities improve with nostalgic stimulation. Comparing the experimental and control groups during the nostalgic intervention, the older adults group demonstrated significantly enhanced orientation, memory, and fluency compared to the control group.

Moreover, culture is a significant factor influencing multiple dimensions such as cognition, recognition, and expression among the older adults (Makri & Giannouli, 2022) [34]. From a cognitive perspective, seniors in Eastern and Western cultural contexts exhibit distinct differences: those in Western-dominated cultures tend to prioritize rational analysis and logical reasoning, while those in Eastern cultures lean more toward emotional analysis and contextual understanding. From a psychopathological perspective, culture exerts differential influences on mental disorders—Western depression is more likely to manifest as somatization, whereas Eastern dementia symptoms tend to align more closely with Alzheimer’s disease. Culture also exerts distinct influences across socioeconomic strata and generations. Consequently, this study exhibits cultural specificity, and its findings cannot be extrapolated beyond their contextual backdrop. This suggests that future research should incorporate cultural dimensions as variables in study designs. Furthermore, subsequent evaluations and formulations of aging-friendly strategies should be refined by integrating underlying cultural contexts.

### Theoretical contributions

First, integrating nostalgia theory with cognitive experiments in older adults offers a novel perspective for understanding changes in emotional interactions within this demographic. Second, within the broader societal context of actively embracing aging, this study pioneers the application of nostalgia therapy in cognitive intervention processes, thereby broadening avenues for non-pharmacological intervention research. Furthermore, this study adopted a theory-practice integration approach, leveraging existing resources and field survey data to interpret the relationship between nostalgic contexts and cognitive levels in older adults, thereby providing a theoretical foundation for cognitive strategy research in this population.

### Practical implications

This study offers insights for planning community senior activity areas and nursing homes. Incorporating nostalgic elements into community designs can enhance the emotional well-being and cognitive function of older adults. It also provides strategic pathways for investors and designers of community facilities and nursing homes. Furthermore, it encourages nursing home administrators to collaborate with local communities through regular nostalgia therapy activities, such as vintage film screenings, to further promote the physical and mental health of older adults.

### Limitations and future research

First, the pilot study had a small sample size and lacked follow-up data, making it impossible to determine the sustainability of nostalgia-stimulated effects. The content requires further optimization. During questionnaire surveys and interviews, data may be subject to potential social desirability bias, as participants sometimes unconsciously select socially acceptable responses. The testing process involves extensive recall elements that are highly subjective and difficult to quantify, potentially leading to bias. Future studies could employ longitudinal comparisons to reduce such errors. Additionally, this study suffers from gender sample limitations. The higher proportion of female participants may skew results toward female groups. Future research should incorporate more balanced gender samples to ensure the universal applicability of the nostalgia empowerment effect. Finally, the data analysis did not categorize participants by economic status or physical health conditions, which may have influenced the findings.

## Conclusions

### Key findings

This study confirms that cognitive fluency is key to overcoming the “last mile” challenge in cultural consumption. By activating emotional identification through nostalgia and enhancing activity participation through culturally adapted technologies, this approach warrants greater attention in future research on cognitive enhancement strategies for older adults.

### Future directions

Future research can further optimize the user experience of immersive technologies like VR for older adults. Building upon the comprehensive evaluation framework for immersive environments proposed by Wang and Zhang (2025), this provides a solid theoretical foundation for constructing a quantitative assessment system. Through mixed-method research, Wang and Zhang identified system quality, institutional support, and personal innovation as key determinants of user experience outcomes in immersive technology applications. Building upon this foundation, future evaluation frameworks should incorporate dimensions like system quality while also addressing the psychological and physiological conditions of older adults users. Emphasis should be placed on mitigating issues such as dizziness and visual fatigue during the technological experience, pursuing more natural and adaptive virtual interactions. This provides a more scientific and concrete evaluation framework and development direction for the aging-friendly evolution of technologies like VR.

Furthermore, optimizing ethical standards is indispensable. Establishing a more robust ethical framework should prioritize the local storage of sensitive data such as activity trajectories and electroencephalograms, while allowing the older adults population to autonomously determine the scope of data sharing. This approach enables further exploration of dynamically optimizing ethical norms while safeguarding individual privacy.

## Data Availability

The raw data supporting the conclusions of this article will be made available by the authors, without undue reservation.
